# Effect of electrical cardioversion on 1‐year outcomes in patients with early recurrence after catheter ablation for atrial fibrillation

**DOI:** 10.1002/clc.23663

**Published:** 2021-06-08

**Authors:** Zheng Dong, Xin Du, Xiao‐Xia Hou, Liu He, Jian‐Zeng Dong, Chang‐Sheng Ma

**Affiliations:** ^1^ Department of Cardiology, Beijing Anzhen Hospital Capital Medical University, National Clinical Research Center for Cardiovascular Diseases Beijing China; ^2^ Cardiovascular Center, Beijing Tongren Hospital Capital Medical University Beijing China; ^3^ Department of Cardiology The First Affiliated Hospital of Zhengzhou University Zhengzhou China

**Keywords:** atrial fibrillation, catheter ablation, electrical cardioversion, recurrence, rehospitalization

## Abstract

**Background:**

Atrial fibrillation (AF) recurrence is common in the 3‐month blanking‐period after catheter ablation, during which electrical cardioversion (ECV) is usually performed to restore sinus rhythm. Whether ECV can affect the clinical outcome of post‐ablation AF patients is inconsistent, however. We aimed to explore the 1‐year effect of ECV on AF recurrence and rehospitalization in patients experienced recurrence within 3‐month after AF catheter ablation.

**Methods:**

Patients who experienced recurrence within 3‐month after AF catheter ablation procedure were enrolled from the China Atrial Fibrillation Registry (China‐AF). A 1:3 Propensity score matching (PSM) method was applying to adjust the confounders between patients who had been treated by ECV or not. Logistic regression models were conducted to evaluate the association of ECV with 1‐year AF recurrence and rehospitalization.

**Results:**

In this study, 2961 patients experienced AF recurrence within 3‐month after the procedure, and 282 of them underwent successful ECV, 2155 patients did not undergo ECV. One‐year AF recurrence rates were 56.4% in ECV group versus 65.4% in non‐ECV group (*p* = .003), and were 55.9% versus 65.9%, respectively, after PSM (adjusted odds ratio [OR] 0.66; 95% confidence interval (CI): 0.49–0.88, *p* = .005). However, the difference of 1‐year rehospitalization rates between two groups were not statistically significant before (ECV group: 23.7% vs. non‐ECV group: 22.3%, *p* = .595) and after PSM (ECV group: 24.4% vs. non‐ECV group: 21.6%, adjusted OR1.14; 95% CI 0.81–1.62, *p* = .451).

**Conclusions:**

Successful ECV was associated with lower rate of one‐year recurrence in patients with early recurrent AF after catheter ablation.

## 1 INTRODUCTION (BACKGROUND)

Atrial fibrillation (AF) is the most common sustained arrhythmia in clinical practice. Its incidence increases dramatically with age and underlying heart disease. It is recognized as a global public health problem due to its significant burden of morbidity and mortality.^1^, ^2^, ^3^, ^4^

Catheter ablation of AF is effective in restoring and maintaining sinus rhythm. Nowadays, it is gradually being widely used to treat AF improving the overall symptom burden and quality of life.^5^, ^6^, ^7^, ^8^ Although its efficacy has been established, arrhythmia recurrences thereafter still remain an important issue. AF recurrence rates have been reported in up to 50% of patients in the early 3‐month after catheter ablation.^5^, ^8^ The current expert consensus statement recommends that early recurrence of atrial arrhythmias in a 3‐month “blanking period” should not be considered as ablation failure, and repeat ablation should not be performed routinely, except in special cases.^5^ Pharmacological cardioversion and electrical cardioversion (ECV) are commonly used in patients with early recurrent AF following ablation procedure.^7^, ^8^ However, the effects of antiarrhythmic drugs are unpredictable, with high rate of adverse effects.^9^ ECV is an efficient and safe procedure to restore sinus rhythm, with high‐initial success rate, but the long‐term maintenance of sinus rhythm remains a challenge.^10^, ^11^, ^12^

Whether restoration of sinus rhythm by ECV in patient with early recurrent AF after catheter ablation is associated with improved clinical outcomes remains unclear. Our study aimed to evaluate the association between successful ECV and clinical outcomes in AF patients with early recurrence after catheter ablation using data from the China Atrial Fibrillation Registry (China‐AF) study.

## 2 METHODS

### 2.1 Study population

We used data from the China‐AF study. It is a prospective, multicenter, hospital‐based, on‐going registry of patient with AF from 31 hospital in Beijing, China. From August 2011 to December 2018, a total of 25 512 patients were enrolled. The Human Research Ethics Committee at Beijing Anzhen Hospital approved this study and the ethics review boards at individual participating hospitals approved their participation. Written informed consent was obtained from each patient. Details of this study have been described previously.^13^

Eligible patients were aged ≥18 years, with AF documented via either electrocardiogram (ECG) or Holter monitoring and followed‐up for at least 12 months. These patients should receive AF catheter ablation upon registry and have recurrence of AF within 3 months after ablation. Exclusion criteria were as the followings: (1) patients having other diseases with life expectancy <1 year; (2) patients with transient AF caused by reversible cause (e.g., cardiac surgery, pulmonary embolism, untreated hyperthyroidism); (3) valvular AF; (4) patients undergoing AF catheter ablation again or surgical ablation within 3 months after AF catheter ablation.

All patients underwent extensive encircling pulmonary vein (PV) isolation to isolate the PVs. Other additional ablations, such as linear ablation and/or complex fractionated arterial electrogram ablation, depended on the operator's judgment. Oral anticoagulants were administrated for at least 3 months after catheter ablation. Those patients with recurrent AF after ablation were performed ECV depending on the decision of the physician according to guidelines.^7^, ^8^

### 2.2 Data collection

Information on sociodemographic characteristics (age, gender, education status, and medical insurance coverage); body mass index (BMI); smoking and drinking status; AF type (newly diagnosed, paroxysmal or persistent); time of AF diagnosis; echocardiographic measurements (left atrial diameter [LAD]; left ventricular ejection fraction [LVEF]); previous ablation history; medical history including chronic heart failure (CHF), hypertension, diabetes mellitus (DM), established coronary artery disease (CAD), previous stroke/transient ischemic attack (TIA)/peripheral thromboembolism (TE), as well as medication history including antiarrhythmic drugs (AADs), oral anticoagulant drugs, rate‐control drugs were collected when patients were enrolled. BMI was calculated by dividing weight in kilograms by the square of height in meters. Current smoking was defined as regular smoking within 1 month and at least one cigarette per day, and current drinking was defined as regular drinking within 1 month and at least 50 g of alcohol each time. Established CAD was defined as having any history of myocardial infarction, percutaneous coronary intervention, or coronary artery bypass grafting. AADs included class I (propafenone, moricizine, mexiletine) and class III (amiodarone, sotalol). Rate‐control drugs included β‐blocker, non dihydropyridine calcium antagonists and digoxin. CHA_2_DS_2_‐VASc was calculated by assigning 2 points for a history of stroke/TIA/TE and age ≥ 75 years, 1 point for age between 65 and 74 years, a history of hypertension, DM, congestive heart failure, vascular disease, and female sex.

Patients were followed‐up at 3 months, 6 months, and every 6 months thereafter by trained staff, at the outpatient clinics or through telephone interview. Information relating to medical therapies, Holter electrocardiography, repeat ECV, repeat catheter ablation, surgical ablation, pharmacologic cardioversion and rehospitalization were collected at each follow‐up occasion.

### 2.3 Study outcomes

The primary and secondary clinical outcome was one‐year incidence of AF recurrence and rehospitalization after catheter ablation, respectively.

In this study, AF recurrence was defined based on the symptoms reported by the patients or the record of AF/atrial flutter (AL)/atrial tachycardia (AT) documented on the Holter electrocardiography during the follow‐up period. Patients who performed repeated catheter ablation or surgical ablation or repeated ECV or pharmacologic cardioversion due to AF/AL/AT during follow‐up were also considered to have AF recurrence. ECV was deemed successful if sinus rhythms were continuously recorded for at least 1 hour after the shock. Pharmacologic cardioversion included oral or intravenous administration AADs due to AF/AL/AT. Rehospitalization included hospitalization for all reasons except for repeat ECV.

### 2.4 Statistical analysis

A matched cohort was constructed between ECV patients and Non‐ECV patients. Each case of ECV was matched to 3 non‐ECV patients by propensity score matching (PSM). The matched variables considered for the PS analysis were presented in Table 1. Continuous data were presented as mean ± standard deviation analyzed by Student's t‐test in normal distribution, or median (25–75th percentiles) analyzed by Wilcoxon rank sum test in non‐normal distribution. Categorical data were presented as numbers and percentages (%), analyzed by Chi square test. Univariate and multivariate logistic regression analyses were used to compare rates of AF recurrence and rehospitalization between groups, as well as calculate the odd ratio (OR) and 95% confidence interval (CI). Potential confounders should be adjusted including antiarrhythmic drugs, rate‐control drugs during follow‐up for the analysis of AF recurrence. As for AF rehospitalization, oral anticoagulants during follow‐up period should be considered. Subgroup analysis was conducted to explore the differential effects of ECV on the AF recurrence by age, sex, AF type, duration of AF, BMI, LAD, CHF, established CAD and CHA_2_DS_2_‐VAS_C_. Analyses were also performed for subgroups to explore the differential effect of ECV on the rehospitalization with age, sex, health insurance, education level, AF type, established CAD and CHA_2_DS_2_‐VAS_C_. A 2‐sided *p* value <.05 was considered to be statistically significant. All analyses were performed using SAS version 9.4 (SAS Institute Inc., Cary, North Carolina).

**TABLE 1:** Baseline characteristics of patients by ECV usage

Characteristics	Before PSM				After PSM			
	Overall	ECV	Non‐ECV	*p*	Overall	ECV	Non‐ECV	*p*
	2397	282	2115		1016	254	762	
Age (years) mean (SD)	58.98 (10.40)	58.44 (10.86)	60.04 (10.43)	.016	59.85 (10.49)	58.65 (10.80)	59.09 (10.27)	.559
<65, n (%)	1582 (66.0%)	202 (71.6%)	1380 (65.3%)	.034	723 (71.2%)	183 (72.1%)	540 (70.9%)	.719
≥65, n (%)	815 (34.0%)	80 (28.4%)	735 (34.8%)		293 (28.8%)	71 (28.0%)	222 (29.1%)	
Male, n (%)	1599 (66.7%)	202 (71.6%)	1397 (66.1%)	.062	733 (72.2%)	186 (73.2%)	547 (71.8%)	.657
Education level								
High school or below, n (%)	1659 (69.1%)	189 (67.0%)	1467 (69.4%)	.424	695 (68.4%)	169 (66.5%)	526 (69.0%)	.459
University or above, n (%)	741 (30.9%)	93 (33.0%)	648 (30.6%)		321 (31.6%)	85 (33.5%)	236 (31.0%)	
Health insurance								
Yes, n (%)	1738 (72.5%)	188 (66.7%)	1550 (73.3%)	.019	748 (73.6%)	186 (73.2%)	562 (73.8%)	.869
No, n (%)	659 (27.5%)	94 (33.3%)	565 (26.7%)		268 (26.4%)	68 (26.8%)	200 (26.3%)	
BMI (kg/m^2^) mean (SD)	26.47 (3.50)	26.62 (3.58)	25.98 (3.68)	.010	26.47 (3.50)	26.62 (3.58)	26.42 (3.48)	.423
<28, n (%)	1581 (73.9%)	175 (68.9%)	1406 (74.6%)	.053	702 (69.1%)	175 (68.9%)	527 (69.2%)	.938
≥28, n (%)	558 (26.1%)	79 (31.1%)	479 (25.4%)		314 (30.9%)	79 (31.1%)	235 (30.8%)	
Current smoking, n (%)	508 (21.2%)	55 (19.5%)	362 (17.1%)	.320	193 (19.0%)	51 (20.1%)	142 (18.6%)	.612
Current drinking, n (%)	417 (17.4%)	71 (25.2%)	437 (20.7%)	.081	269 (26.5%)	68 (26.8%)	201 (26.4%)	.902
AF type								
Newly diagnosed, n (%)	35 (1.5%)	5 (1.8%)	30 (1.4%)	<.001	23 (2.3%)	5 (2.0%)	18 (2.4%)	.882
Paroxysmal, n (%)	1473 (61.5%)	103 (36.5%)	1370 (64.8%)		350 (34.5%)	90 (35.4%)	260 (34.1%)	
Persistent, n (%)	889 (37.1%)	174 (61.7%)	715 (33.8%)		643 (63.3%)	159 (62.6%)	484 (63.5%)	
Duration of AF (years) mean (SD)								
<1, n (%)	820 (34.2%)	110 (39.0%)	710 (33.6%)	.071	379 (37.3%)	93 (36.6%)	286 (37.5%)	.793
≥1, n (%)	1577 (65.8%)	172 (61.0%)	1405 (66.4%)		637 (62.7%)	161 (63.4)	476 (62.5%)	
LAD (mm) mean (SD)	42.41 (5.60)	42.79 (5.46)	40.27 (5.71)	<.001	42.41 (5.60)	42.96 (5.28)	42.33 (5.69)	.091
<45, n (%)	1986 (82.9%)	207 (73.4%)	1779 (84.1%)	<.001	744 (73.2%)	183 (72.1%)	561 (73.6%)	.624
≥45, n (%)	411 (17.2%)	75 (26.6%)	336 (15.9%)		272 (26.8%)	71 (28.0%)	201 (26.4%)	
History of AF ablation, n (%)	207 (8.6%)	24 (8.5%)	183 (8.7%)	.936	88 (8.7%)	23 (9.1%)	65 (8.5%)	.797
Medical history, n (%)								
Heart failure	136 (5.7%)	25 (8.9%)	111 (5.3%)	.014	81 (8.0%)	20 (7.9%)	61 (8.0%)	.947
Hypertension	1277 (53.3%)	143 (50.7%)	1134 (53.6%)	.358	557 (54.8%)	135 (53.2%)	422 (55.4%)	.536
Diabetes mellitus	505 (21.1%)	66 (23.4%)	439 (20.8%)	.306	228 (22.4%)	58 (22.8%)	170 (22.3%)	.862
Previous stroke/TIA/TE	255 (10.6%)	25 (8.9%)	230 (10.9%)	.304	105 (10.3%)	23 (9.1%)	82 (10.8%)	.439
Established CAD	262 (10.9%)	24 (8.5%)	238 (11.3%)	.166	95 (9.4%)	24 (9.5%)	71 (9.3%)	.950
Antiarrhythmic drugs usage, n (%)	1260 (52.6%)	158 (56.0%)	1102 (52.1%)	.215	612 (60.2%)	155 (61.0%)	457 (60.0%)	.767
Rate‐lowering drugs usage, n (%)	660 (27.5%)	69 (24.5%)	591 (27.9%)	.220	275 (27.1%)	68 (26.8%)	207 (27.2%)	.903
Antithrombotic drugs usage, n (%)	2049 (85.5%)	238 (84.4%)	1811 (85.6%)	.582	912 (89.8%)	228 (89.8%)	684 (89.8%)	1.000
CHA_2_DS_2_‐VAS_C_, mean (SD)^a^	1.747 (1.3)	1.68 (1.2)	1.84 (1.3)	.005	1.75 (1.3)	1.69 (1.2)	1.77 (1.3)	.289
0–1, n (%)	1166 (48.6%)	157 (55.7%)	1009 (47.7%)	.012	523 (51.5%)	141 (55.5%)	382 (50.1%)	.137
≥2, n (%)	1231 (51.4%)	125 (44.3%)	1106 (52.3%)		493 (48.5%)	113 (44.5%)	380 (49.9%)	

Abbreviations: AF, atrial fibrillation; BMI, body mass index; CAD, coronary artery disease; ECV, electrical cardioversion; LAD, left atrial diameter; PSM, propensity score matching; TIA, transient ischemic attack; TE, thromboembolism.

a CHA_2_DS_2_‐VAS_C_ as calculated by assigning 2 points for a history of stroke/TIA/thromboembolism and age ≥ 75 years, 1 point for age between 65 and 74 years, a history of hypertension, DM, congestive heart failure, vascular disease and female sex.

## 3 RESULTS

### 3.1 Baseline characteristics

**FIGURE 1:**
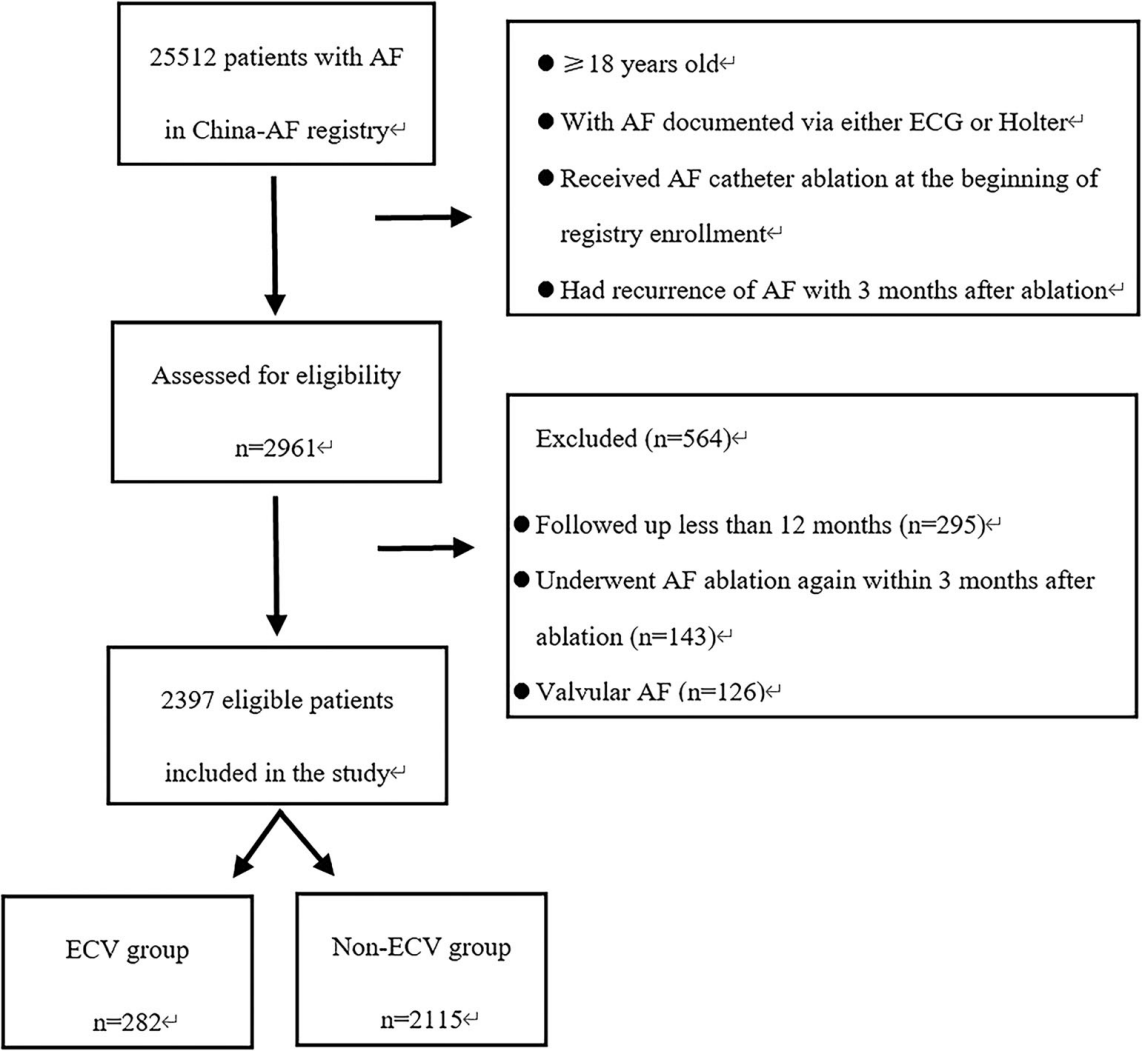
Flowchart of patients. AF, atrial fibrillation; ECG, electrocardiogram; ECV, electrical cardioversion

In the present study, 12 090 patients received AF catheter ablation upon registry. Of them, 2961 patients (24.5%) had AF recurrence within 3 months and 2666 patients were followed‐up for at least 12 months. 143 patients underwent re‐ablation within 3 months after ablation and 126 patients with valvular fibrillation were exclude from the analyses. Finally, a total of 2397 patients were eligible for this study, 282 patients underwent successful ECV within 3 months (ECV group), 2115 patients did not undergo ECV within 3 months (non‐ECV group) (Figure 1).

The baseline characteristics in ECV and non‐ECV groups before and after PSM were listed in Table 1. Before PSM, patients in ECV group were younger (58.4 vs. 60.0 years, *p* = .016), more likely to be persistent AF (61.7% vs. 33.8%, *p* < .001) and more likely to have CHF (8.9% vs. 5.3%, *p* = .014). Unmated patients in ECV group also had higher BMI (26.62 vs. 25.98 kg/m^2^, *p* = .010), larger left atrial size (42.79 vs. 40.27 mm, *p* < .001), but the score of CHA_2_DS_2−_VASc (1.68 vs. 1.84, *p* = .012) was lower. While after matching, all the baseline characteristics between ECV and non‐ECV groups were no significant differences.

### 3.2 Clinical outcomes

#### 3.2.1 AF recurrence analyses

**FIGURE 2:**
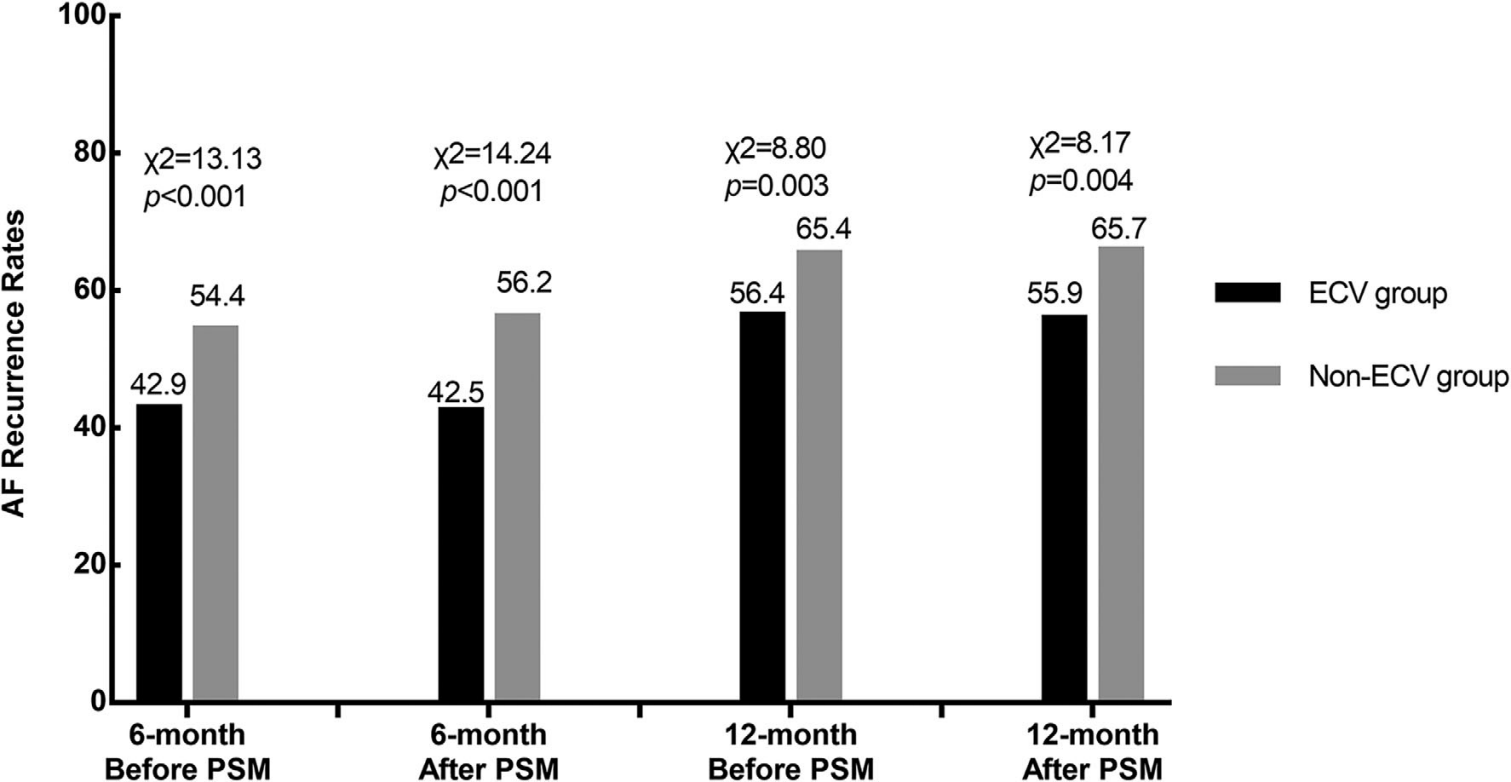
AF recurrence rates in patients during follow‐up before and after PSM. AF, atrial fibrillation; ECV, electrical cardioversion; PSM, propensity score matching

**FIGURE 3:**
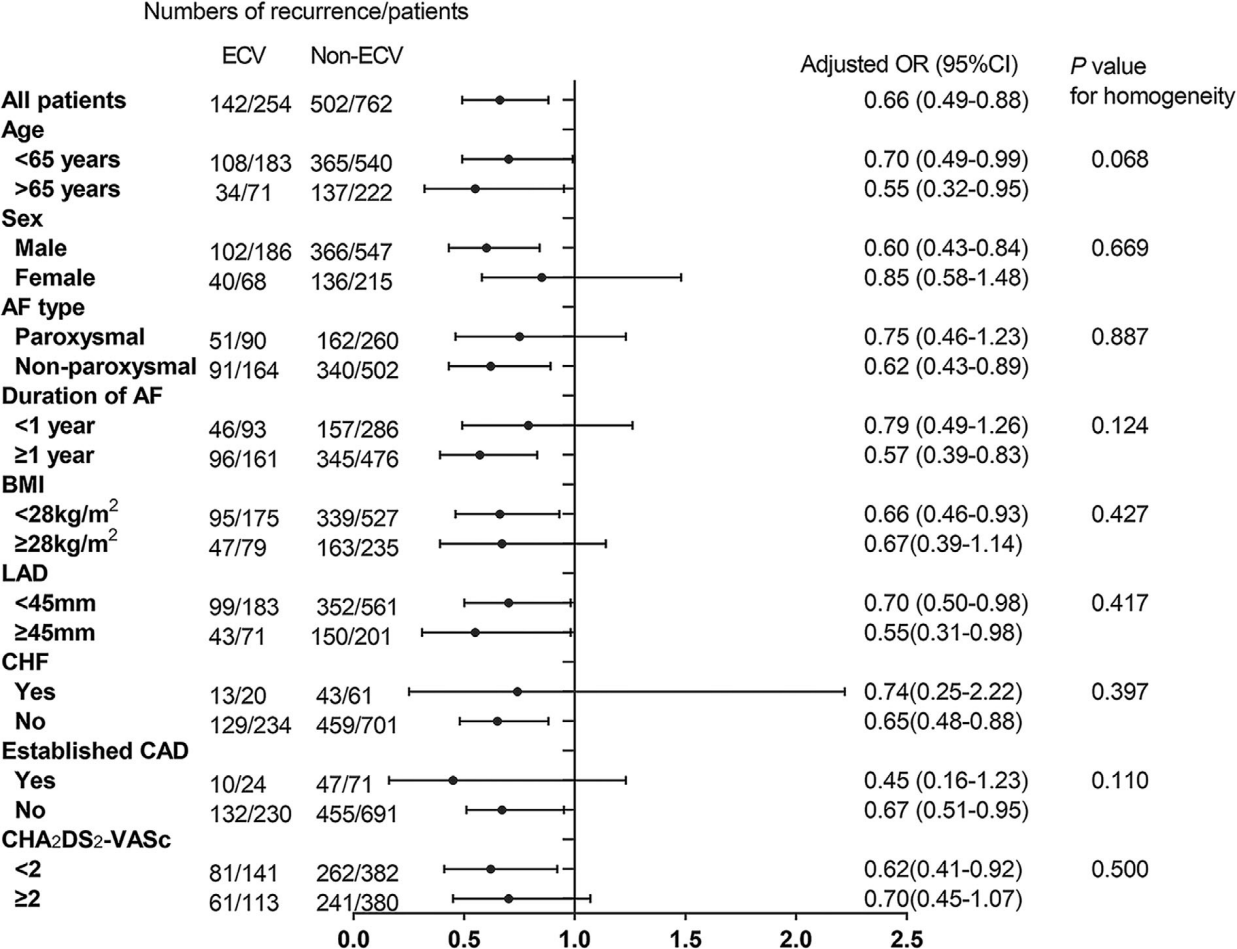
Subgroup analysis for AF recurrence rates in patients with and without ECV. Models were adjusted for antiarrhythmic drugs and rate‐control drugs during the follow‐up. AF, atrial fibrillation; BMI, body mass index; CAD, coronary artery disease; CHF, chronic heart failure; ECV, electrical cardioversion; LAD, left atrial diameter

Before PSM, the AF recurrence rates were 56.4% in ECV group and 65.4% in non‐ECV group for one‐year (*p* = .003), 42.9% in ECV group and 54.4% in non‐ECV group for 6‐month (*p* < .001), respectively. After PSM, they were 55.9% in ECV group and 65.9% in non‐ECV group for one‐year (*p* = .004), 42.5% in ECV group and 56.2% in non‐ECV group for 6‐month (*p* < .001), respectively (Figure 2). Compared to non‐ECV group, the OR of AF recurrence in ECV group was 0.66 (95% CI 0.49–0.89, *p* = .004) in univariate logistic regression analysis. In multivariate logistic regression analysis, it was 0.66 (95%CI 0.49–0.88, *p* = .005). We found that the ORs of ECV and AF recurrence were homogenous within different subgroups (Figure 3).

#### 3.2.2 AF rehospitalization analyses

Before PSM, 25 patients (8.9%) in ECV group and 45 patients (2.1%) in non‐ECV group experienced rehospitalization for repetitive‐ECV (χ2 = 39.84, *p* < .001), while, 24 patients (9.5%) in ECV group and 18 patients (2.4%) in non‐ECV group after PSM (χ2 = 24.14, *p* < .001).

**FIGURE 4:**
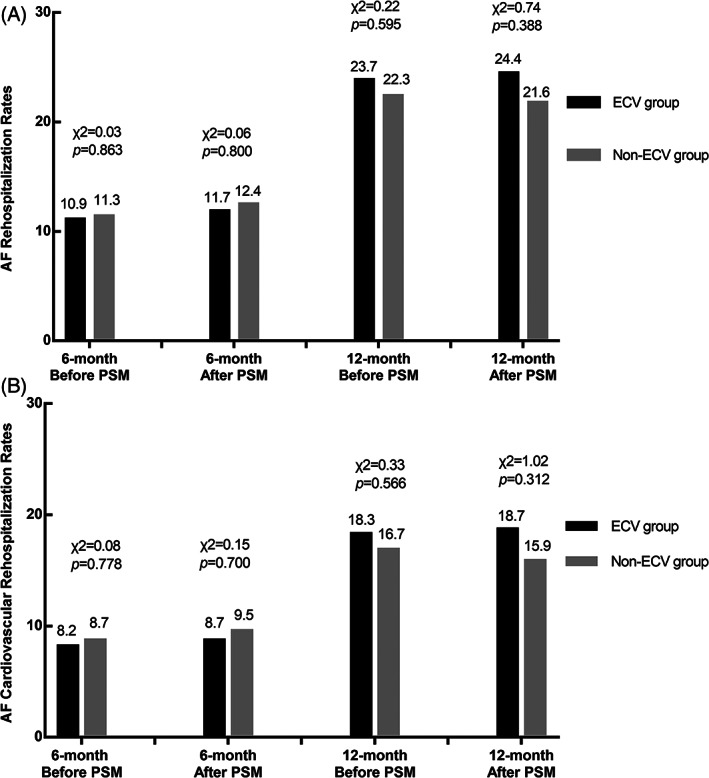
AF rehospitalization rates (A) and cardiovascular rehospitalization rates (B) in patients during follow‐up before and after PSM. AF rehospitalization rates (A) in patients during follow‐up before and after PSM. Cardiovascular rehospitalization rates (B) in patients during follow‐up before and after PSM. AF, atrial fibrillation; ECV, electrical cardioversion; PSM, propensity score matching

**FIGURE 5:**
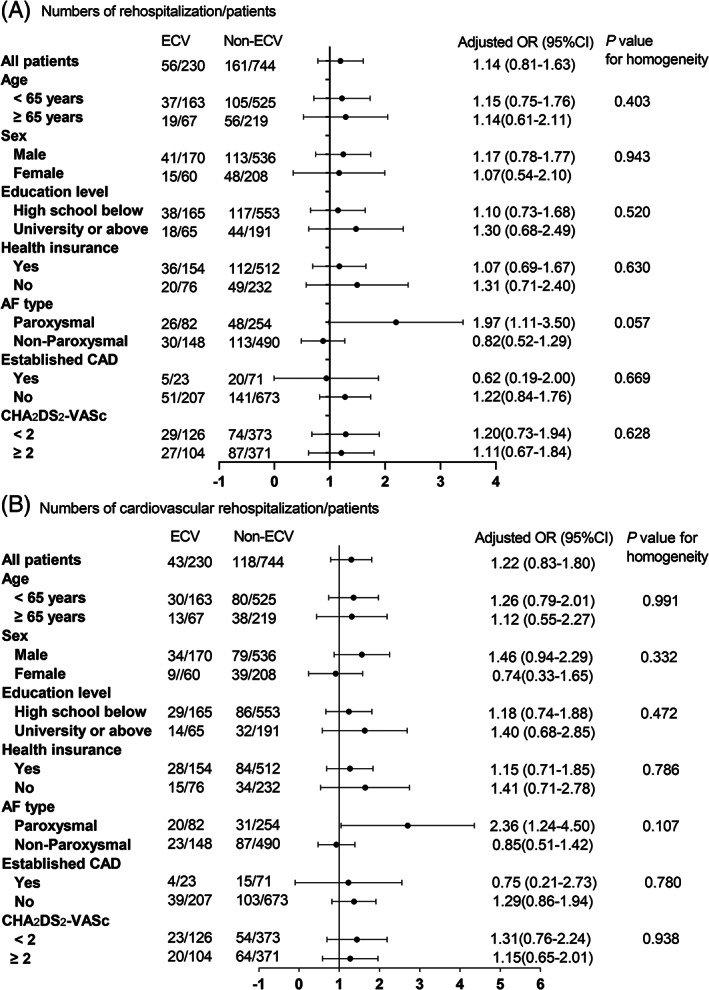
Subgroup analysis for AF rehospitalization rates (A) in patients with and without ECV. Models were adjusted for antiarrhythmic drugs, rate‐control drugs and anticoagulant drugs during follow‐up. AF, atrial fibrillation; CAD, coronary artery disease; ECV, electrical cardioversion

Before PSM, 61 patients (23.7%) experienced rehospitalization in ECV group and 461 patients (22.3%) in non‐ECV group for 1‐year (*p* = .595), 28 patients (10.9%) in ECV group, and 233 patients (11.3%) in non‐ECV group for 6‐month (*p* = .863), respectively. After PSM, 56 patients (24.4%) experienced rehospitalization in ECV group and 161 patients (21.6%) in Non‐ECV group for 1‐year (*p* = .388), 27 patients (11.7%) in ECV group and 92 patients (12.4%) in non‐ECV group for 6‐month (*p* = .863), respectively (Figure 4(A)). Compared to non‐ECV group, the OR of AF rehospitalization in ECV group was 1.17 (95%CI 0.82–1.65, *p* = .389) in univariate logistic regression analysis, and 1.14 (95%CI 0.81–1.63, *p* = .451) in multivariate logistic regression analysis. The ORs of ECV and rehospitalization were homogenous within subgroups, but in the subgroup analysis of AF type, there was a critical statistical significance (Figure 5(A)). 23 patients (10.0%) underwent re‐ablation in ECV group during one‐year follow‐up, while 61 patients (8.2%) in non‐ECV group (χ2 = 0.72, *p* = .395) after PSM.

Before PSM, the 1‐year cardiovascular rehospitalization rates were 18.3% in ECV group and 16.9% in non‐ECV group (χ2 = 0.33, *p* = .567). After PSM, they were 18.7% in ECV group and 15.9% in Non‐ECV group (χ2 = 1.02, *p* = .312) (Figure 4(B)). Compared to non‐ECV group, the OR of AF rehospitalization in ECV group was 1.22 (95%CI 0.83–1.79, *p* = .312) in univariate logistic regression analysis, and 1.22 (95%CI 0.83–1.80, *p* = .310) in multivariate logistic regression. The ORs of ECV and cardiovascular rehospitalization were homogenous within subgroups (Figure 5(B)).

## 4 DISCUSSION

In this large contemporary cohort of Chinese patients with non‐valvular AF, we found that ECV was associated with lower recurrence rate during one‐year follow‐up in patients with early recurrent AF after catheter ablation. We observed no relationship between ECV and rehospitalization or cardiovascular rehospitalization at 1 year, also in the subgroup analysis.

Previous studies^5^, ^14^reported that early recurrence rates of AF after catheter ablation variated between 16% and 65%, and might be related to transient inflammatory reaction resulting from ablation lesions or pericarditis. Other possible mechanisms include a transient imbalance of the autonomic nervous system, which being an arrhythmia trigger, and a reconnection of PVs.^15^, ^16^, ^17^ AF causes electrical and structural changes driving the atrial remodeling process.^3^, ^18^, ^19^ The proinflammatory phase following ablation also favors to the maintenance of AF.^20^ When AF persists, the progression of AF is facilitated to result in fibrosis and enlargement of left atrium. The reduction of AF burden could initiate a long‐term process of reverse remodeling.^3^, ^21^ Some studies supporting rapid functional and structural remodeling during AF encourage the clinician to administer cardioversion early in patients with recurrence after AF ablation.^3^, ^5^ So, we believe that maintenance of sinus rhythm during the “blanking period” after ablation is beneficial to improve long‐term outcomes.^18^ In clinical practice, we usually administer AADs, ECV to restore sinus rhythm in these patients. Patients who experience early recurrence of AF are more likely to have persistent AF in the long run,^14^, ^22^, ^23^, ^24^ approximately 30%–50% of patients with an early recurrence may later remain free from recurrent atrial arrhythmias.^18^, ^23^, ^25^, ^26^ Therefore, early repeat ablation is not recommended routinely in patients with early recurrence after ablation.^5^ Additionally, despite the use of AADs, it may suppress nonspecific arrhythmias and reduce hospitalization in the immediate post‐ablation period, but it does not affect the underlying disease course in the long term.^14^, ^22^, ^27^, ^28^ Early ECV after AF catheter ablation appears effective to maintain sinus rhythm, minimize late AF recurrence, reduce chronic AADs use, and avoid re‐ablation procedures.

Previous studies have failed to reach a consistent conclusion as to whether ECV usage in patients with early AF recurrence after ablation was associated with the reduction of AF recurrence. The reason for this inconsistency is unclear but may be related to study population, definition of AF recurrence, follow‐up protocol and statistical methods. Contrary to our findings, in a study conducted by Johns Hopkins Hospital, Chilukuri et al.^29^ reported the outcomes of patients who underwent cardioversion for persistent AF/AL following ablation. They showed that not only >80% of patients who underwent cardioversion for persistent AF/AL after AF ablation had recurrence, but also early or late ECV (within 90 days or between 90 and 180 days following ablation) did not affect the outcome. Ebert et al.^22^ also failed to reveal that successful early ECV after AF ablation could prevent more recurrence of AF. However, consistent with the findings of our study, in a prospective study,^18^ Malasana et al. demonstrated that patients undergoing their first cardioversion early after ablation (<3 months) were more likely to remain free from arrhythmia at 1 year. An aggressive strategy of rapid cardioversion post‐ablation reduces the significance of recurrent AF/AL and prevent the long‐term need for recurrent ablations or chronic antiarrhythmic medications during the first 6 months. In another retrospective study,^30^ Baman et al. founded that freedom from AF/AL was achieved in approximately 50% of patients who undergo cardioversion within 30 days of a persistent atrial arrhythmia after catheter ablation of AF. Patients who underwent early cardioversion within 30 days of arrhythmia recurrence were >20 times more likely to remain in sinus rhythm than patients who were cardioverted after 30 days, regardless of the timing of recurrence or whether concomitant antiarrhythmic drug therapy was used. Our observation suggested that the usage of ECV to restore sinus rhythm may be associated with the reduction of AF recurrence in the long term for those patients. Early restoration of sinus rhythm after AF recurrence may facilitate the long‐term maintenance of sinus rhythm. Nevertheless, our study patients came from the largest, prospective, multi‐center registered AF study in China, including paroxysmal and Non‐paroxysmal AF. Moreover, AF recurrence in our study include AF, AL, and AT.

Symptom status may be a marker of high risk for hospitalization. Similar to the result of Outcomes Registry for Better Informed Treatment of Atrial Fibrillation (ORBIT‐AF) study,^31^ they revealed patients with more severe symptoms and requiring electrophysiology management were also more likely to experience hospitalization. The Atrial Fibrillation Follow‐up Investigation of Rhythm Management (AFFIRM) study^32^ pointed out that cardioversion was a maker of lower quality of life and greater symptom burden. They found that hospitalization rates were higher in patients with a rhythm control strategy relative to rate control (80% vs. 73%, *p* < .001). Pokorney et al.^33^ also demonstrated that patients that received cardioversion were at high risk of rehospitalization and symptomatic progression of AF. The reason may be when patients with symptomatic AF recur after cardioversion, they may be more aware of the impact of AF on their quality of life. Patients without cardioversion had adapted to the limitations of their symptoms over time. In our study, the usage of ECV in patients with early recurrence after AF ablation could not be associated with lower rates of rehospitalization and cardiovascular rehospitalization. Moreover, in the subgroup analysis, rehospitalization appeared to be higher in patients with paroxysmal AF. Patients requiring ECV usually have more severe symptoms, exertional limitation, and poor tolerance. Patients with paroxysmal AF also have more obvious symptom. These reasons lead to more rehospitalization and require more aggressive rhythm control. Contrary to our findings, Tripathi et al.^2^ reported ECV during index admission was associated with a significant reduction in 30‐day readmissions and service charges. This may be partly due to the heterogeneity of the study samples (our study samples were patients with early recurrent AF after catheter ablation), partly due to the period of study time (the duration of our study was 1‐year).

Among the patients included in our study, most of them were younger male with insurance, so they were more likely to be actively hospitalized for rhythm control therapy. In agreement with our study, Patel et al.^34^ showed marked increases in AF hospitalization among those ages 35–64 years, possibly due to more aggressive inpatient treatments such as AF ablation, direct current cardioversion in this younger population. Although patients are hospitalized more frequently and treated with more costly and aggressive inpatient therapies, but this is associated with improved outcomes including decreased rates of in‐hospital mortality and long‐term mortality.^4^

## 5 STRENGTHS AND LIMITATIONS

The China‐AF registry is the largest prospective cohort in China and these data were from clinical practice, our results were certainly applicable and instructive. However, this was an observational study and should be considered hypothesis generating. Despite an aggressive PSM technique, residual unmeasured confounders may influence the findings. Patients with asymptomatic AF or other under‐reporting situations, such as insufficient continuous ambulatory rhythm or ECG monitoring might affect results of our study. Although the China‐AF study population was large, the proportion of patients with ECV was relatively low, which may have an effect on the outcome. Lastly, the follow‐up time period of 1‐year was relatively short.

## 6 CONCLUSIONS

Our study showed that successful ECV was associated with a lower rate of 1‐year recurrence in patients with early recurrent AF after catheter ablation, but not associated with the rates of rehospitalization and that for cardiovascular rehospitalization.

## CONFLICT OF INTEREST

Changsheng Ma has received honoraria from Bristol‐Myers Squibb (BMS), Pfizer, Johnson & Johnson, Boehringer‐Ingelheim (BI), and Bayer for giving lectures. Jianzeng Dong has also received honoraria from Johnson & Johnson for giving lectures. Other authors have no disclosures.

## Data Availability

Data available on request due to privacy/ethical restrictions
